# Book Notices

**Published:** 1895-04

**Authors:** 


### Book Notices.

A Clinical Manual of Diseases of the Eye, Including a Sketch of its Anatomy. By D. B. St. John Roosa, M. D., LL. D., Professor of Diseases of the Eye and Ear in the New York Post-Graduate Medical School and Hospital; Surgeon to the Manhattan Eye and Ear Hospital; formerly Professor of Diseases of the Eye in the University of the City of New York, and in the University of Vermont; Consulting Surgeon to the Brooklyn Eye and Ear Hospital; President of the New York Academy of Medicine; Honorary Member of the Medico-Chirurgical Society of Edinburgh; Honorary Fellow of the

Academy of Medicine of Havana, Cuba, etc., etc. Octavo, 650 pages, 178 engravings in the text, nearly all original, two full-page chromo-lithographic plates, and a full-page black plate. Bound in extra muslin at $5.50, and in sheep at $6.50. William Wood & Company, Publishers, 43, 45 and 47 East Tenth Street, New York City.

This book is divided into four parts. Part I, embracing five chapters, treats of the anatomy and physiology of the eye and its appendages. Part II also contains five chapters, and discusses the relative frequency of different diseases of the eye, methods of examination, therapeutics, and surgery of the eye. Part III, which contains fifteen chapters, treats of diseases of the eyelids, the lachrymal apparatus, the conjunctiva, eyeball, and orbit. In Part IV, the conditions of the eye requiring the use of glasses, errors of refraction and accommodation, strabismus, and affections of the ocular muscles, are carefully considered in eight chapters.

The author's aim was not to prepare a cyclopedic text-book of ophthalmology, but to furnish the practitioner with a book that contains a complete and safe guide for the management of cases with eye diseases. The author's large experience in the treatment of diseases of the eye, and his well-known easy and interesting style of writing, have served as contributing factors in the production of this valuable work.

In part first, a good description of the anatomy and physiology of the various parts of the eye and its appendages,—eyebrows, eyelids, muscles, tarsal cartilages, cilia, blood vessels, conjunctiva, cornea, uveal tract, choroid, retina, optic nerves, etc., is given. This part of the book is unusually rich in illustrations.

In part second, the relative frequency of different diseases of the eye is discussed. This part also explains the methods of examining the eye, including the use of the ophthalmoscope and the ophthalmometer. The principles of treatment and remedies applicable, and surgical operations on the eye, are included in this part of the book.

Part third treats of diseases of the eye and its appendages in the following order: The eyelids, the lachrymal apparatus, the conjunctiva, the cornea, the sclera, the iris, the ciliary region, the choroid, the retina, the vitreous humor, the optic nerve, glaucoma, the crystalline lens, the orbit.

In part fourth, which considers errors of refraction and accommodation, strabismus, and affections of the ocular muscles, the author differs from many of the leading ophthalmologists, and expresses his convictions in no unmistakable terms. He thinks that too much stress has been put upon the condition of the eyes in producing constitutional symptoms.

He teaches that there are no reflex symptoms from the eyes, unless the eyes themselves give warning that they are not doing their work properly. He thinks that the profession generally have committed a grave error in believing that any morbid constitutional condition, of more than a passing nature, can be dependent upon an error of refraction, or a latent insufficiency of an external muscle of the eye. The practice of referring cases suffering with migraine, vertigo, chorea, etc., but without any ocular symptoms, to the oculist, and their treatment for ocular defects in the hope of relieving the head symptoms or neuroses, he regards as unjustifiable and useless. He cites a number of cases suffering with head symptoms, some of whom had been under the care of oculists for a long period of time, and who were finally cured by appropriate treatment directed to the general system, no treatment whatever having been directed to the eyes.

The book is thoroughly practical, contains much that is valuable, not only to the specialist, but to the general practitioner as well. H.

---

A Synopsis of the Practice of Medicine for Practitioners and Students. By William Blair Stewart, A. M., M. D., Lecturer on Therapeutics; late Instructor on Practice of Medicine in the Medico-Chirurgical College of Philadelphia; Demonstrator in the Philadelphia School of Anatomy, etc. One large octavo volume, about 434 pages, cloth, $2.75. E. B. Treat, Publisher, 5 Cooper Union, New York.

This book, which is a carefully prepared synopsis of the Practice of Medicine, is intended to give to the busy practitioner and student, at a small cost, concise and accurate information on the etiology, symptomatology, pathology, diagnosis, prognosis and treatment of disease.

It is not intended as a substitute for the more voluminous works on this subject, for they have their proper uses, but as a ready reference work when the physician's time for study is limited, and especially when he wishes to refresh his memory on the most important points in practice, and at the same time secure information fully up to date, this volume will serve an excellent purpose.

The book is not only carefully written, but good judgment has been exercised in leaving out that which is of the least importance that the volume might be of convenient size and the discussions brief.

It is printed on fine heavy paper, and handsomely bound; in fact, both the author and publisher have done their work in a highly creditable manner. H.

---

NOTES ON THE NEWER REMEDIES, THEIR THERAPEUTIC APPLICATIONS AND MODES OF ADMINISTRATION. By David Cerna, M. D., Ph. D., Demonstrator of Physiolgy, and Lecturer on the History of Medicine in the Medical Department of the University of Texas; formerly Assistant in Physiology, Demonstrator of, and Lecturer on, Experimental Therapeutics in the University of Pennsylvania, etc., etc., etc. Second edition enlarged and revised. Cloth, 250 pages. Price $1.25. W. B. Saunders, Publisher, 925 Walnut Street, Philadelphia.

This little volume furnishes the physician with much reliable information not easily obtained elsewhere. In fact, it would be impossible to find in any other single volume the amount of real information concerning the newer drugs found in this.

The author does not attempt to make any classification, but takes up the newer remedies in alphabetical order, giving their physical properties, solubility, therapeutic applications, administration, and chemical formula. The facts are stated in the briefest possible way, thus permitting the discussion of the greatest number of subjects in the smallest amount of space. The book forms a very valuable addition to the various works on therapeutics, and the condensed form in which it is prepared makes of it a convenient book of reference. The author has given especial attention to therapeutics, and the practical value of the book is very much increased thereby. H.

---

A MANUAL OF BANDAGING, Adapted for Self-Instruction. By C. Henry Leonard, A. M., M. D. Professor of the Medical and Surgical Diseases of Women and Clinical Gynæcology in the Detroit College of Medicine. Sixth edition, with 130 engravings. Cloth, octavo, 139 pages. Price, $1.50. The Illustrated Medical Journal Co., Publishers, Detroit, Mich.

The main feature for commendation of this book over other similar works is that each illustration shows the direction of the various turns of the bandage with arrow-heads, and each turn is properly numbered; this renders the book a self-instructor by the reader of it, who has but to put the various bandages about the limbs of an office companion a few times: when the "trick" of its application upon a patient has been learned. It takes the place, in this way, of hospital drill. Besides the "Roller Bandages,,' the various Ts "Cravats," "Slings," "Tailed," "Adhesive" and "Plaster" bandages, and "Immovable Dressings" are given. The book is divided into sections treating of "The Bandages of the Head," of "The Body," of "The Upper Extremity," of "The Lower Extremity," "Knots," "Strappings," "Compresses" and "Poultices" with full description of making and applying the same. There is an illustration for nearly every bandage described. It has been recommended as a text book in various medical colleges and hospitals in this country, and has had two editions sold abroad. A medical student could profitably spend his vacation evenings in mastering the application of bandages by using this book as a guide, and to a practitioner it would not come amiss.

---

SYLLABUS OF GYNECOLOGY, Based on the American Text-book of Gynecology. By J. W. Long, M. D., Professor of Gynecology and Pediatrics in the Medical College of Virginia, etc., etc. 131 pages. Cloth. W. B. Saunders, 925 Walnut St., Philadelphia. $1.00 net.

This syllabus has been arranged in conformity with the American Text-book of Gynecology, and will be highly appreciated by those who have read that book. The subjects are arranged in tabular form, and the author has not hesitated to differ from or add to the Text-book whenever, in his judgment, it was best to do so.

The syllabus will be especially valuable to the medical student in attendance on lectures. The outline of the lecture will be before him, and as blank space is left for the purpose, he can make such additional memoranda as seems to him of the greatest importance. It will also serve as a ready reference for the physician and will assist him in many cases in making a diagnosis.

H.

---

HYDRIODIC ACID AND THE HYPOPHOSPHITES, THERAPHEUTICAL INDICATIONS AND CLINICAL DATA. By R. W. Gardner, 158 William Street, New York. Twelfth edition. 194 pages. Cloth.

Hydriodic acid is one of our most important therapeutic agents, and we welcome any information concerning it. Mr. Gardner was the pioneer in its manufacture, and in its introduction to the medical profession, hence what he has to say on this subject will be received with more than the ordinary interest. He has devoted much time and care to the preparation of this little volume, and it will doubtless meet with much favor at the hands of the medical profession.

Among the contributors to the book are Drs. W. Gill Wylie, John V. Shoemaker, Prof. Germain See, and many others. The subject of the hypophosphites, their therapeutic application, mode of administration, etc., is included in this volume.

To anyone interested in these subjects Mr. Gardner proposes to furnish a copy of the book free upon application to him for it.

---

A COMPEND OF THE PRACTICE OF MEDICINE: Including a very complete section on Skin Diseases, and a new section on Mental Diseases. By Daniel E. Hughes, M. D., Demonstrator of Clinical Medicine at the Jefferson Medical College, Philadelphia, etc. Physician's Edition (two compend volumes in one), 568 pages, morocco-gilt binding, $2.50. P. Blakiston, Son & Co. Philadelphia: 1895.

This valuable work has reached its fifth edition, which bespeaks its popularity, as well as the author's efforts to keep it fully up to the recent advances made in medicine.

An intermediate book is needed, between a compend and a treatise on the practice of medicine, and Dr. Hughes' work fulfills that want, and he presents a volume equally valuable to the student and the busy practitioner. His long experience with quizzing students for the U. S. Army examinations, and his large clinical experience makes his book unusually complete for one of its size. It presents the latest ætiology of diseases, and the most recent treatment, which makes it invaluable to the profession. The author devotes much space to skin diseases, and appends a section on mental diseases, which is constantly demanding more attention upon the part of the general practitioner. The work being bound in flexible morocco, with gilt edges, makes it an ornament to any library. M. M. S.

---

ESSENTIALS OF CHEMISTRY AND TOXICOLOGY—For the Use of Students in Medicine. By R. A. Witthans, A. M., M. D., Professor of Chemistry and Physics in the University of New York; Professor of Chemistry and Toxicology in the University of Vermont, etc. Twelfth edition; 314 pp. Price, cloth, $1.00. William Wood & Co., publishers, 43, 45, 47 E. 10th street, New York City.

This little volume is prepared especially for the student and practitioner of medicine. It will be found of every day use to the physician, as it treats fully of physiological chemistry, which is one of the most important foundations of rational medicine. Much attention has also been directed to the chemistry of therapeutics, while the chemisty of pharmacy has been passed by without notice. The book is short and practical, and will serve the purpose for which it was intended most admirably. H.

---

LABORATORY GUIDE FOR THE BACTERIOLOGIST, by Langdon Frothingham, M. D. V., Assistant in Bacteriology and Veterinary Science, Sheffield Scientific School, Yale University. Illustrated. Price, cloth, 75 cents. W. B. Saunders, Publisher, 925 Walnut St., Philadelphia. 1895.

This book is arranged as conveniently and concisely as possible and will be found a great convenience to the student of bacteriology in his laboratory work. Heretofore it has been necessary to refer to the large text-books and search through many pages of matter in order to find directions for the preparation and examination of bacteriological specimens. Here we have this information in one convenient little volume. H.

---

WHERE TO SEND PATIENTS FOR WATER CURES AND CLIMATIC TREATMENT. By Dr. Thomas Linn, Doctor of Medicine, Faculty of Paris; Doctor of Medicine and Surgery, University of New York, etc., etc. 44 pages, paper cover. London: Henry Kimpton, 82 High Holborn, W. C.; Hirschfield Bros., Bream's Building, Fetter Lane, E. C.

This little volume furnishes definite information regarding the European mineral waters and health resorts; the special advantages of each in various diseased conditions, the climatic conditions, etc. It also contains an alphabetical table giving a list of maladies, with names of the best places to send patients to in Europe. H.

